# PneumoKITy: A fast, flexible, specific, and sensitive tool for *Streptococcus pneumoniae* serotype screening and mixed serotype detection from genome sequence data

**DOI:** 10.1099/mgen.0.000904

**Published:** 2022-12-14

**Authors:** Carmen L. Sheppard, Sam Manna, Natalie Groves, David J. Litt, Zahin Amin-Chowdhury, Marta Bertran, Shamez Ladhani, Catherine Satzke, Norman K. Fry

**Affiliations:** ^1^​ Vaccine Preventable Bacteria Section, Respiratory and Vaccine Preventable Bacteria Reference Unit, UK Health Security Agency, London NW9 5EQ, UK; ^2^​ Translational Microbiology Group, Murdoch Children’s Research Institute, The University of Melbourne Department of Paediatrics at the Royal Children’s Hospital, Parkville, Victoria, Australia; ^3^​ Respiratory and Vaccine Preventable Bacteria Reference Unit, UK Health Security Agency, London NW9 5EQ, UK; ^4^​ Immunisation and Vaccine Preventable Diseases, UK Health Security Agency, London NW9 5EQ, UK; ^†^​ Present address: Genpax Ltd, 9 Pembridge Road, Notting Hill, London, W11 3JY, UK

**Keywords:** bioinformatics, carriage, colonisation, detection, epidemiology, Pneumococcus, serotyping, software

## Abstract

Determination of serotypes of *Streptococcus pneumoniae* is essential for monitoring current vaccine programmes. Since October 2017, pneumococcal serotypes in England have been derived from whole genome sequencing (WGS) data using our bioinformatic tool PneumoCaT. That tool was designed for serotype determination from pure cultures in a reference laboratory. To help determine multiple serotypes in pneumococcal carriage samples, we developed a new software tool named PneumoKITy (Pneumococcal K-mer Integrated Typing) that uses the powerful Mash k-mer screening method for pneumococcal serotyping. Mash k-mer screening is more sequence specific and much faster than the mapping method used in PneumoCaT and can determine 54 (58.1  %) of the 93 serotypes in the SSI Diagnostica phenotypical serotyping scheme to type level with the remainder called to serogroup or subgroup level (e.g., 11A/D). PneumoKITy can be run on both FastQ and assembly input, requiring up to 11× less memory and running up to 29× faster than the current version of PneumoCaT (1.2.1) on FastQ files. PneumoKITy can be used as a rapid, flexible serotype screening method which adds sensitive detection of mixed serotypes, e.g., for nasopharyngeal carriage studies where the presence of multiple serotypes is common. PneumoKITy’s ability to function from assembly file, for pure culture serotype detection, increases its speed. This speed potentially enables the software to be run using low infrastructure overhead via web-based platforms. PneumoKITy could be used as a fast initial screening method with other tools used for those serotypes that could not be fully determined to type level if necessary. PneumoKITy was found to be highly accurate and sensitive when run on a panel of FastQ files derived from mixed cultures with all serotypes in 47/51 (92.2  %) of samples being accurately detected. PneumoKITy was also able to accurately estimate the relative abundance of serotypes in the same sample. Estimates being within a mean relative abundance of 1.5 % of the expected abundance in mixtures with known concentrations. PneumoKITy was able to detect minor serotypes with expected abundance of 1 % in the known mixture serotypes. PneumoKITy is a rapid, flexible tool with wide-ranging applications outside of the pure-culture, reference laboratory serotyping remit of PneumoCaT.

## Data Summary

PneumoCaT development and validation genomic datasets can be obtained on the European Nucleotide Archive (ENA) at project PRJEB14267. Genomic data from Public Health England (PHE), now UK Health Security Agency (UKHSA), pure culture carriage isolates used for validation of the method are available at ENA under project PRJEB34491.

Genomic data from known mixture (simulated mixed carriage) can be obtained from National Centre for Biotechnology Information (NCBI) Sequence Read Archive under project PRJNA561126.

The data used in this manuscript are listed in Table 1 and Fig. S1 and Tables S1–S6 https://doi.org/10.6084/m9.figshare.21067339.v1 [1].

PneumoKITy is available on Github at https://github.com/CarmenSheppard/PneumoKITy.

**Impact Statement**

We describe a new bioinformatic tool, PneumoKITy, for determination of *Streptococcus pneumoniae* serotype or serogroup from genomic data. PneumoKITy offers sensitive screening of multiple serotypes in complex samples and is computationally fast and efficient. Sensitive multiple serotype detection is important for detection of low abundance serotypes of pneumococcus circulating in the population, and their potential impact on future vaccination policies. PneumoKITy offers flexible run options and can be optimised for either serotype screening from expected pure or expected mixed culture input. In pure run mode PneumoKITy, uniquely, can be used on both FastQ and genome assembly input data or, if run in mix mode, can screen FastQ data for detection of multiple serotypes. The run time for one genome assembly in pure culture detection mode is milliseconds, and in all run modes PneumoKITy offers reduced run-time and computational load compared to existing methods. While PneumoKITy lacks the fine serotyping determination for some serogroups offered by other methods, it has application in the efficient screening of large datasets by reducing computational load in conjunction with other methods to subtype samples that cannot be distinguished fully using PneumoKITy alone and offers sensitivity for detection and quantification of mixed serotypes in complex samples.

## Introduction

*Streptococcus pneumoniae* (the pneumococcus) is an important bacterial pathogen that can also be carried asymptomatically in the nasopharynx. A systematic analysis of the global burden of disease in 2016 found that *S. pneumoniae* was the leading cause of disease, causing 197 million episodes of lower respiratory infection globally and 1 189 937 deaths, more than all the other aetiologies combined [2]. Effective vaccines have been introduced and licenced for use since 2000. However, vaccine-induced immune responses are specific to the capsular polysaccharide (CPS) that surrounds the bacterial cell and are limited in serotype valency, with existing vaccines only targeting up to 23 serotypes while over 100 distinct serotypes of pneumococcus have been described [3].

In 2006, the publication of the full CPS operon sequences of 90 serotype reference strains [4], enabled the development of sequence-based methods for serotyping. PneumoCaT (Pneumococcal Capsular Typing) published in 2016 [5] was the first fully automated pneumococcal serotyping method available that offered comprehensive serotyping for a reference laboratory, enabling 94.7 % of the serotypes that could be defined using the gold standard serotyping sera (SSI Diagnostica) to be distinguished using whole genome sequencing. In October 2017 this method was implemented into the routine *S. pneumoniae* reference service at Public Health England (PHE), now UK Health Security Agency (UKHSA).

There has been increasing interest in recent years in methods for accurate detection of multiple serotypes carried in the nasopharynx. Studies have demonstrated that multiple serotype carriage is common [6–8] and methods to sensitively detect serotypes occurring at lower abundance in carriage have been devised and compared [9] as use of these methods would increase the knowledge of circulating serotypes and their potential impact when considering vaccination policy and vaccine development.

Although PneumoCaT is an accurate method for serotype discrimination from whole genome sequencing (WGS) data, it is not particularly fast to run, taking up to several minutes per serotype determination on a standard computing infrastructure and does not have the necessary sensitivity for detection of multiple serotypes required for studies of more complex samples (e.g., nasopharyngeal swabs) rather than pure cultures (e.g., from CSF or blood samples).

Other serotyping methods have been developed using variations of the PneumoCaT Capsular Type Variant database (CTVdb) such as seroBA [10], which offers faster serotyping from FastQ, and SeroCall [11], which provides mixed serotype detection from FastQ data. Although SeroBA offers higher speed serotyping, it does not offer any advantage in mixed serotype detection over PneumoCaT, and indeed may be more limited in detection of mixed types due to the partially assembly-based methods used. Although SeroCall offers highly accurate mixed serotype detection, the use of mapping for serotype screening increases the run time over the kmer-screening method used by SeroBA.

We have developed PneumoKITy (Pneumococcal K-mer Integrated Typing), a lightweight tool using a fast k-mer-based method for pneumococcal serotype/serogroup screening. This method offers speed, input datatype flexibility and increased sensitivity to mixed serotypes over the original PneumoCaT tool. PneumoKITy can be run with either FastQ or assembly input data for expected pure culture detection which gives more limited ability to detect mixed serotypes, or for expected mixed serotype detection with enhanced sensitivity (FastQ data only). PneumoKITy offers a sensitive, accurate, flexible, and fast alternative which can be useful for initial screening of large datasets with lower computational overhead, from either assemblies or FastQ data or for detection of serotype mixtures from FastQ files.

## Methods

### Definitions

For the purposes of this manuscript – the following definitions are used throughout.

**Serotype** – the serologically determined type of the organism (phenotype), e.g., 6B.

**Serogroup** – a group of serotypes that are related serologically using phenotypical typing sera, e.g., serotype 6A, 6B, 6C and 6D form serogroup 6.

**Genogroup** – a group of strains that have capsular operon sequences that are related to each other, e.g., serotypes 12A, 12B, 12C, 12F, 44, and 46 are considered within the same genogroup due to their capsular operon sequence similarity.

**Genetic subtype** – a detectable genetic difference between strains that are considered the same phenotypical serotype, e.g., genetic type 23B1 which is a previously described subtype of serotype 23B [12].

### Reference sequences and information

As a source of reference sequence information and for benchmark comparisons, PneumoCaT version 1.2.1 was downloaded from the Github repository (https://github.com/phe-bioinformatics/PneumoCaT). The reference file from the PneumoCaT 1.2.1 CTVdb for the stage 1 operon mapping ‘reference.fasta’ was used as the basis for much of this work. This reference file contains 95 CPS operon sequences including those from [4] and some extra references and capsular variants as detailed in [5]. Gene sequence reference files for serogroups that had stage 2 gene presence/absence or allele variants from the CTVdb were also used. The yaml files (‘mutationdb.yml’) from the PneumoCaT CTVdb folders for each genogroup, containing serotype-specific gene variants for all serotypes determined in stage 2 by gene presence/absence or allele determinations, were used to assemble the information for the new CTVdb SQL database used in PneumoKITy.

### Bacterial sequences and standard bioinformatic processing

The sequences used for analysis are noted in Table 1. All isolates except the mixed serotype sequences had been analysed as part of the standard PHE/UKHSA genomic pipeline as discussed in Kapatai *et al*. [5], which includes quality trimming of the Illumina reads, species identity check, multi-locus sequence typing (MLST) and PneumoCaT 1.0 serotyping.

**Table 1.** Isolates or sequences used for analysis

Isolate/sequence	no. of isolates/genomes	Study reference or supplier	Comments
**Reference isolates**	92	SSI Diagnostica	Bacterial type strains of 92 different serotypes used for creating serotyping sera. Genomic data for these isolates are included in the PneumoCaT development set [5] ENA PRJEB14267 (See Table S1)
**PneumoCaT validation panel genomes**	2054	[5]	Validation panel. Genome data from PneumoCaT original validation ENA PRJEB14267 (seeTable S2 for list) [5]
**Nasopharyngeal carriage isolates**	876	[23]	Isolates from large studies of pneumococcal nasopharyngeal carriage [23], ENA PRJEB34491 See Table S3.
**Serogroup 6 and 19 Subtypes capsular operon sequences**	44	[16]	Subtype sequences from a paper on the diversity of serogroup 6 and 19 CPS operons [16] GenBank, accession numbers JF911487–JF911531
**Mixed serotype samples**	60	[11]	Illumina reads from pneumococcal co-cultures or serotypes mixed in known concentrations [11]. Bioproject accession number PRJNA561126

See https://doi.org/10.6084/m9.figshare.21067339.v1 for Supplementary tables.

### Initial bioinformatic and data analysis methods

Marbl Mash version 2.3 [13] was used to assess the k-mer distance between reference capsular operon sequences. Mash Dist was used to create a non-tabular distance matrix comparing the reference sketch files. Using default settings, k-mer length 21 and max 1000 non-redundant k-mers. Serotypes that were too closely related using Mash Dist would fall into genogroups that would need further serotype determination using specific gene variants (stage 2). The resulting distance data were used to determine whether the Mash method could be viable, and to inform the potential new genogroup configurations.

As initial proof of principle, Mash Screen [14] was used to screen FastQ files obtained from 91 type strain isolates (SSI Diagnostica) against the CPS reference sequences, sketched using Mash Sketch with default parameters, which were k-mer length 21 (-k) and max non-redundant k-mer numbers 1000 (-s) (subsequently referred to as ‘k-mer number’). The data were filtered to remove non-match values, arbitrarily assigned as any with shared k-mer hash values less than 40 % of the total hashes, to reduce the large number of data points for analysis. The percentage hits were then calculated (shared hashes/total hashes × 100) and this metric is used throughout this manuscript.

Analysis of the Mash Screen input parameters was assessed using Mash Sketch to produce various versions of the sketched reference sequence fasta file, with k-mer length parameters (-k) 15, 21, 31 and in later experiments k-mer numbers (-s) 1000 and 25 000 as input values for the references.

Sequence identity, median multiplicity and p-values reported by Mash were assessed for usefulness in the context of screening CPS operon sequences.

After initial assessment of the Mash Screen parameters, the conformation of the new genogroups of serotypes that were appropriate to the new method and that would be included in the new CTVdb were established.

### Development of PneumoKITy software and CTVdb

PneumoKITy software was written in Python version 3.9 [15], Python packages Pandas 1.0.3 and Numpy 1.21.5 were used for data analysis within the software and SQLalchemy 1.3.10 was used for interactions with the CTVdb for interpretation of the Mash Screen results.

The software has two different run modes depending on whether the input sample data is from an expected pure culture isolate (referred to as ‘pure’ run mode) or expected mixed culture (referred to as ‘mix’ run mode). This changes the parameters and outputs to suit each type of run.

Mash Screen was used for all screening analyses and is run as a sub-process in the Python [15] software. The fasta file of the CPS operon references was sketched using Mash Sketch on the command line, with option -i to allow sketching of multiple references from the multi-fasta into one ‘references.msh’ sketch file. This reference_msh file of sketched sequences became the overall reference sequence set for the new programme. Input FastQ or assembly data are screened by Mash in the same way, the only difference being for assembly data, detection of mixed serotypes is very insensitive due to mixtures of sequences being lost in the assembly process.

A new SQLite3 CTVdb was designed to hold information regarding the phenotypical serotype, genetic types, stage 1 Mash serotype hits, variants, genogroups and genes. Information from the PneumoCaT 1.2.1 CTVdb and documentation was assembled in Microsoft Excel and modified according to the newly assigned genogroups (see Results) and the structure of the tables in the database. Phenotype was separated from genotype to enable both a genetic type and a predicted phenotypical type to be reported based on interpretation data stored in the CTVdb to allow a potential future update where this facility for reporting genetic type separately from predicted phenotype might be used more widely. The subtype operon references for serogroups 6 and 19 (Table 1) [16] were included in the new CTVdb to improve sensitivity for detection of these variable genogroups in stage 1 and enable a genetic subtyping method to be added to future versions of software. The final database consisted of seven linked tables containing unique information.

A RED-AMBER-GREEN (RAG) traffic light style system for assessing result quality was implemented based on metrics such as stage 1 hit percentage and stage 2 gene detections hits, with differences based on whether pure or mix run type was used, for example, mixed samples are automatically flagged AMBER when run in pure culture mode but not when run in mix mode.

For each sample run (whether assembly or FastQ input and in both run modes), the CTVdb is used to interpret the hits received in the stage 1 Mash Screen. If no hits are found with the initial percentage hit cut-off (default 90 %) the software reduces the cut-off by 10 % and re-scores the sample until a minimum of 70 % before reporting as a non-typeable or variant organism (e.g., genetic serotype variant) with AMBER RAG status. If no reference operons give a hit above 70 % then the software flags the result as RED. If there is <20 % hit the software alerts the user that the isolate may be acapsular (does not produce a capsule), non-pneumococcal species or the sequence quality may be poor.

At stage 2, analysis for serotypes where the second stage variant types included in the original PneumoCaT 1.2.1 CTVdb that were gene allele and presence, or absence, was added as these types of variants could be analysed using Mash Screen against a reference gene sequence sketch with no need to invoke differing methods. In some cases, only one serotype or sub-genogroups were separated from the overall group due to the specificity of the gene detections used. This method used Mash Screen with the required reference gene sketches (Sketched using Mash sketch and kmer number 1000, kmer length 31) and used the CTVdb to interpret the variants returned. Serotypes 38, 15F, 12F, 15A, 19A and variant 19AF (determined as phenotype 19F) could be fully distinguished from their genogroups, and other serotypes such as 6A/B and 6C/D separated using this method in stage 2 of the PneumoKITy software.

If pure run mode is selected indicating expected pure cultures, but mixed serotypes are determined, the genogroups and estimated abundance of the mixed serotypes are reported but sub-typing is not attempted. However, if mix run mode is selected and mixed serotypes are determined in stage 1, subtyping will be attempted for those subtypes that can be determined using PneumoKITy.

A diagram outlining the flow of the software run for the input options and run modes is shown in Fig. S1 (Fig. S1).

For assessment of PneumoKITy when run on genome assembly files, Unicycler version 0.4.7 [17] was used to create high quality sequence assemblies from the processed FastQ read files for both the validation and carriage datasets.

R version 4.0.2 [18] was used to analyse data using ggplot2 [19] to create plots.

### Updating the initial reference sequences to suit k-mer screening

After initial testing for specificity and sensitivity the CTVdb was updated by inclusion of three additional references in the stage 1 capsular operon screen to make them more suitable for the greater specificity of k-mer screening by representation of more diverse genotypes of the capsular operon sequences.

DNA sequences for the capsular operon were extracted from 33F and 24F isolates chosen from the UKHSA nasopharyngeal carriage study set (ERR3530303 and ERR3530868 respectively). Due to the failure of the 18F SSI reference strain to match the 18F reference for this serotype with a hit value above 90%, the DNA sequence was extracted from an assembly created from this reference isolate strain.

To retrieve the CPS DNA sequences, firstly the FastQ were assembled using Unicycler as described previously. The sequences were then annotated using Prokka [20] with provision of a reference GenBank file containing the Pneumococcal D39 genome (CP000410.2) and GenBank files for all the 91 serotype reference sequences [4] to ensure correct annotation of the capsular operon genes. The DNA sequence from the beginning of the first coding sequence of the first gene of the capsular operon to the end of the last coding sequence were extracted using BioPython (SeqIO) [21]. To check the sequences, they were aligned with the appropriate serotype 18F, 33F and 24F references from the original PneumoCaT reference.fasta file using Mega X [22].

The sequences were appended to the new references.fasta file created for PneumoKITy, and then this was sketched using Mash Sketch (with input options - i for multi-fasta sketching, -k 31 and default 1000 k-mer number).

The references.msh and references.fasta then replaced the existing files in the CTVdb folder within the PneumoKITy software.

The Microsoft Excel template, contained within the Database_tools folder of the PneumoKITy repository, for importing the CTVdb data was updated to include the new references and the existing CTVdb was deleted and recreated using the PneumoKITy sqlalchemydeclarative.py script. Then the new data were imported from the Excel template using the PneumoKITy import_from_excel.py script (at present no update script exists, but due to the small size of the PneumoKITy database, recreating the CTVdb is fast).

The resulting new CPS reference sequences are available labelled 33F2, 24F2 and 18F in the references.fasta in PneumoKITy CTVdb folder on GitHub.

The panels of reference isolates, carriage isolates and the PneumoCaT validation panel and mixed serotype sequences, were then run as the final dataset to validate the ‘PneumoKITy V1.0’ version of the software.

### Validation of PneumoKITy software

PneumoKITy was tested against the original PneumoCaT Validation panel, and panel of reference genomes from the SSI Diagnostica type strains, and from the nasopharyngeal carriage study panel (Table 1) running both FastQ and assembly inputs. The software was run in the pure run mode and other settings at defaults and asked to collate the results into one file (the optional flag -c was added).

In each validation analysis, the serotypes obtained from PneumoKITy V1.0 were compared with the original PneumoCaT serotype and the phenotypical serotype (obtained by slide agglutination) if available. A matching serotype (or genogroup for those that could not be called to type level) was scored 'one', a mismatching serotype/group with PneumoCaT was scored 'zero'. Isolates that were RAG flagged ‘RED’ by PneumoKITy but matched with a phenotypically acapsular isolate and where PneumoCaT had resulted ‘Failed’ were scored 'one' as they represent isolates highly likely to not have full (or recognised) capsular operons or variants. Sequences for which PneumoCaT had failed to give a result and for which PneumoKITy scored as ‘Less than 70 % hit’ were accepted as a match if there was either no phenotypical data existing for the samples, or if these samples showed non-typeable or other difficulty with serotyping (e.g., auto-agglutination). In some cases, PneumoKITy was able to determine a serotype where PneumoCaT failed to produce a result, in these cases the PneumoKITy result was scored 'zero' or 'one' according to whether it matched the phenotypical laboratory-derived serotype if available and 'zero' if not available.

### Testing the multiple serotype detection ability of PneumoKITy

The ability of PneumoKITy to detect multiple serotypes in mixed samples was determined using a dataset of mixed serotype sequences prepared by Knight *et al*. [11]. These sequences (BioProject accession no. PRJNA561126) consisted of DNA from serotypes mixed with known ratios, and samples grown as co-cultures containing anywhere from two to ten serotypes grown for either 2, 4 or 8 h. For these samples, PneumoKITy was run from the FastQ, using the default parameters in mix run mode.

### PneumoKITy V1.0 performance testing

PneumoKITy V1.0 was benchmarked for speed and memory requirements against SeroCall (installed using conda 4.10.3) and PneumoCaT V1.2.1 in pure run mode on FastQ read files from a serotype 1 (determined in stage 1 only) and serotype 15A (determined in stage 2 using presence/absence variant analysis). PneumoKITy was also run using assemblies of the same files again using pure run mode (representing a technical replicate). Benchmarking was performed in triplicate on an Intel Core i7-7700HQ (2.80GHz x4) laptop running Ubuntu 18.04.6 LTS and with 32 GB RAM. The wallclock run time and memory requirements were assessed by invoking the time command (/usr/bin/time -v). The programmes were run requesting a single thread or four threads for the sub-processes, an output folder was specified in all software, but no additional result collate file was requested from PneumoKITy as none of the other software packages offer a result collation option for collating data from multiple runs. To avoid PneumoCaT V1.2.1 gaining an unfair advantage on run two and three of the 15A sample by reuse of existing output files, the outputs were deleted between runs.

The PneumoKITy code is available on GitHub at https://github.com/CarmenSheppard/PneumoKITy.

## Results

### Proof of principle Mash Distance and Mash Screen analysis

Serotypes that would form genogroups at various Mash Distance cut-offs are shown in Table 2 and compared to the genogroups reported by Kapatai *et al*. [5] for PneumoCaT 1.0. All serotypes not contained in the table were distinguishable below these cut-off values (*N*=30).

**Table 2.** Genogroups assigned by Kapatai *et al*. [5] and with Mash Distance analysis at various cut-offs. Equivalent groupings to those defined by Kapatai *et al*. are indicated in green shading, more specific in purple and less specific in pink. Empty coloured cells indicate that at this cut-off level the serotypes separate completely and do not form a genogroup

Kapatai *et al.*	Mash distance 0.2	Mash distance 0.3	Mash distance 0.4
6A, 6B, 6C, 6D, (6E)*	6A, 6B, 6C, 6D	6A, 6B, 6C, 6D	6A, 6B, 6C, 6D
**7A, 7F**	7A, 7F	7A, 7F	7A, 7F
**7B, 7C, 40**	7B, 7C, 40	7B, 7C, 40	7B, 7C, 40, 24F, 24B, 24A, 48
**9A, 9L, 9V, 9** **N**	9A, 9V	9A, 9V	9A, 9V
	9L, 9 N	9L, 9 N	9L, 9 N
**10A, 10B**		10A, 10B	10A, 10B
**10C, 10F**	10C, 10F	10C, 10F	10C, 10F
**11A, 11B, 11C, 11D, 11F**	11A, 11D	11A, 11D	11A, 11D
	11B,11C	11B,11C	11B,11C
**12A, 12B, 12F, 44, 46**	12A, 12B, 12F, 44, 46	12A, 12B, 12F, 44, 46	12A, 12B, 12F, 44, 46
**15A, 15B, 15C, 15F**	15A, 15F	15A, 15B, 15C, 15F	15A, 15B, 15C, 15F
	15B, 15C		
**18A, 18B, 18C, 18F**	18B, 18C	18A, 18B, 18C, 18F	18A, 18B, 18C, 18F
**19B, 19C**	19B, 19C	19B, 19C	19B, 19C, 19F
**22A, 22F**	22A, 22F	22A, 22F	22A, 22F
**23A, 23B, 23F**	23A, 23F	23A, 23F	23A, 23B, 23F,28A,28F,23B,16F,23B1
**24A, 24B, 24F**	24B, 24F	24A, 24B, 24F	
**25A, 25F, 38**	25A, 25F, 38	25A, 25F, 38	25A, 25F, 38
**28A, 28F**	28A, 28F	28A, 28F	
**32A, 32F**	32A, 32F	32A, 32F	32A, 32F
**33A, 33F, (37)†**	33A, 33F	33A, 33F	33A, 33F, 35C
**33B, 33D**	33B, 33D	33B, 33D	33B, 33D
**35A, 35C, 42**	35A, 35C, 42	35A, 35C, 42	35A, 35C, 42
		35F 47F	35F 47F
**41A, 41F**	41A, 41F	41A, 41F, 31	41A, 41F, 31

*Serotype 6E is a genetic variant only and not a distinguishable phenotypical serotype.

†37 is included in this group in Kapatai *et al*. [5] due to shared operon-like sequences in the genome however the sequence included in the reference file is very distinct and therefore does not cluster with the other serotypes in the Mash Distance analysis.

The reference isolate FastQ files were screened against the reference CPS operons (without additional subtypes) using Mash Screen with default settings (k-mer length 21, k-mer number 1000) and the results were assessed using the genogroups assigned with a Mash Dist. cut-off of 0.3.

**Fig. 1.**
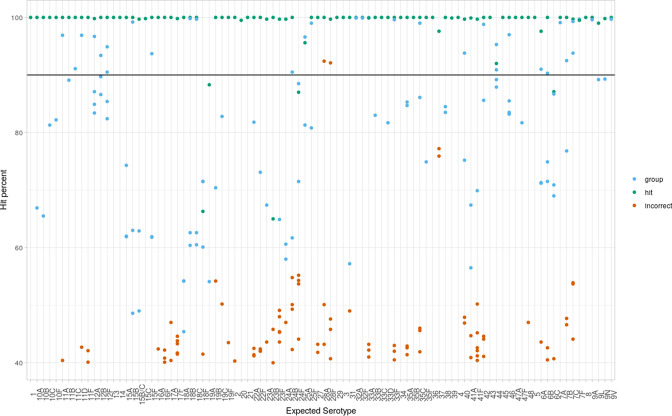
Accuracy of percentage hit (hit kmers/total kmers × 100) for SSI reference strain fastQ against SSI reference operon sequences using Mash Screen with 21 k-mer length and 1000 k-mer number parameters (equivalent to stage 1 analysis with fastQ input). Solid line shows potential 90 % hit cut-off.

Fig. 1 shows the accuracy of all the Mash Screen hits filtered for >40 % (percent hit k-mers/total k-mers). A suggested arbitrary 90 % cut-off line shown was chosen as the starting point for the specificity analysis as it allows for some diversity from the reference sequence.

Top hits for the 6C, 18F, 19A and 24F isolates did not reach the arbitrary 90 % cut-off. Two isolate sequences gave false-positive signals for a serotype that was not the expected serotype or group suggested by the Mash Distance cut-off of 0.03, when a 90 % hit threshold was applied. These two false positive hits were 28F which hit with 28A and 28A which hit with 28F suggesting establishment of a genogroup despite the Mash Distance analysis suggesting they might be separated. The 90 % cut-off was selected as the default due to the high specificity.

### Development of PneumoKITy analysis parameters

An iterative approach to determining the best input analysis parameters for the MASH screen subprocess and subsequent filtering of the Mash Screen result was employed, using the sequences from the reference strains and the full panels of validation and carriage data. The software was developed in stages until the following parameters were adopted.

**Fig. 2.**
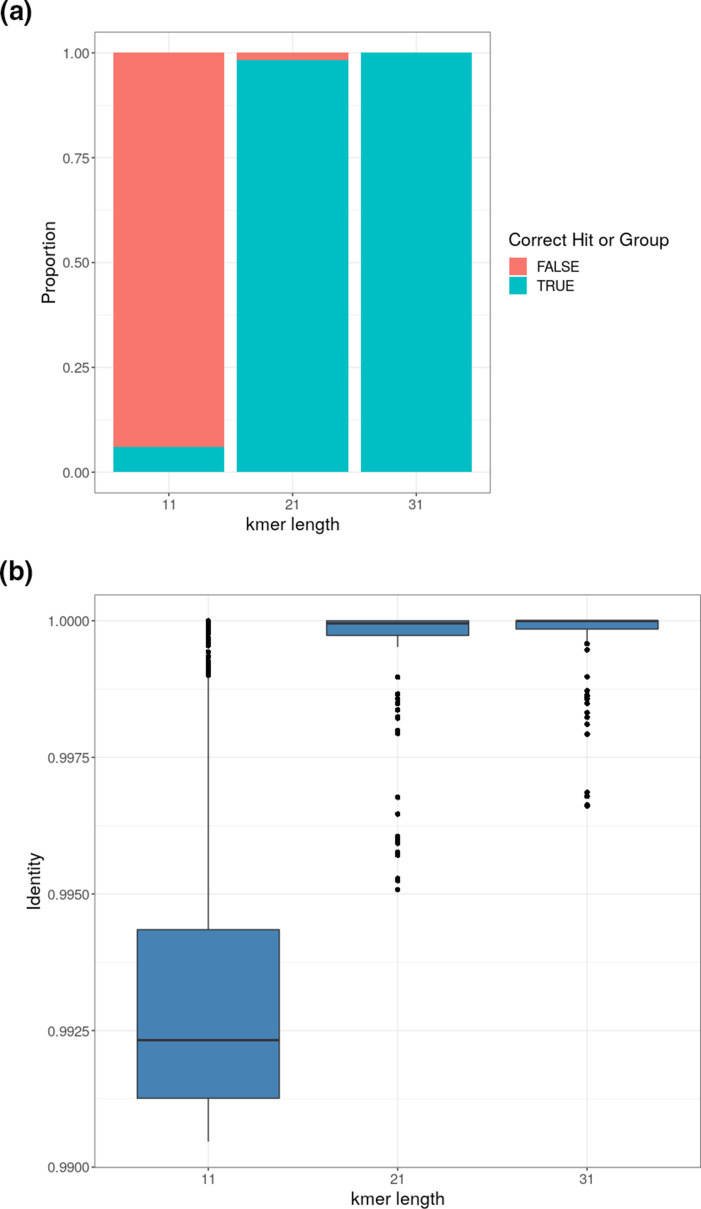
SSI reference strain FastQ screened against SSI reference operon sequences using Mash Screen varying k-mer lengths used for sketch creation. (**a**) Proportion of correct hits ≥90 % hit with differing k-mer lengths. (**b**) Box plot of sequence identity at differing k-mer lengths (a) and (b).

Analysis of k-mer length input value of 31 with initial test k-mer number of 25 000 significantly increased the proportion of hits to the correct serotype or genogroup (as suggested by the previous Mash Distance analysis) above the >90 % suggested percent hit cut-off compared to using the lower setting of 11 (*P*≤ 2.2 x 10^−16^) (Fig. 2). There was not much difference between 21 and 31, but the higher setting was chosen for improved accuracy.

**Fig. 3.**
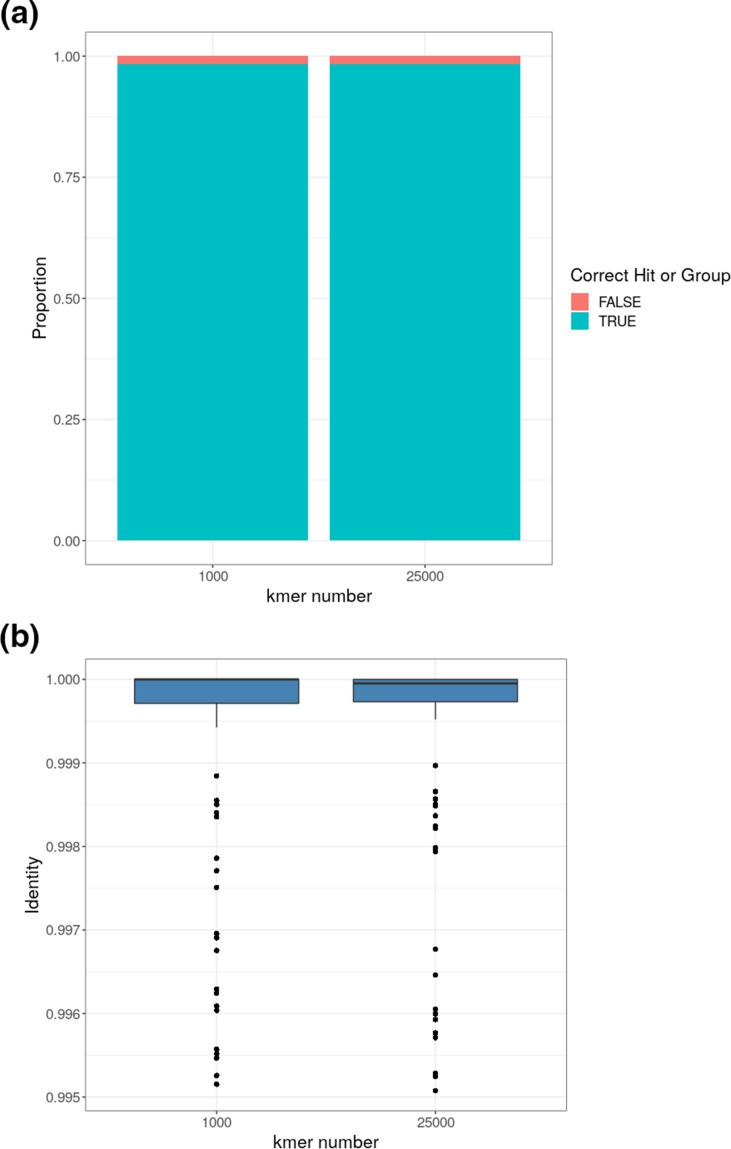
Effect of k-mer number on Mash Screen accuracy and run time for SSI reference isolate FastQ against reference sequences using 90 % hit threshold. (**a**) proportion of correct hits to serotype or group, (**b**) box plot of identity metric (a) and (b).

A comparison of the use of 1000 non-redundant k-mers (k-mer number) vs 25 000 k-mer number with a k-mer length of 31 showed that the proportions of the SSI reference isolates scored correctly was the same (Fig. 3).

Runtime analysis for a serotype 3 FastQ pair run using k-mer number 1000 or 25 000, showed that use of the 1000 k-mer number instead of 25 000, reduced the run time and memory usage from 254.3 s and 957.7 mb to only 29.8 s and 85.6 mb. From these data, max 1000 k-mer number parameter was chosen as this made no difference to the accuracy of the serotype call, but reduced runtime by 65.2 % and memory requirement by 73.4 %.

A combination of 31 k-mer length and 1000 k-mer number parameters were used as final settings for the Mash Sketch or Mash Screen sub-process.

For filtering the output data obtained from Mash Screen *P*-value and median-multiplicity parameters were considered.

*P*-values were all reported as 0 (zero) in the data when filtered for >40 % top hit, representing <2.2×10^−308^ and therefore added no further value for scoring the data, thus these were disregarded as metrics for use in filtering the Mash outcomes.

Median multiplicity was useful for filtering FastQ data and made a difference based on whether pure culture or mixed serotypes were expected in the data. For pure run mode a higher median-multiplicity default cut-off of 10 was employed to avoid oversensitivity to low level contamination. For expected mixed culture inputs (mix run mode) the median-multiplicity default was set to 4.

For assembly input data the median-multiplicity value is not applicable as the data do not generally have multiple k-mer copies, so the median multiplicity cut-off is automatically set to one.

### Determination of genogroups for the CTVdb

The new genogroups from which serotypes can be determined by stage 2 in the PneumoKITy software using either presence or absence or gene allele determinations only are shown in Table 3.

**Table 3.** Genogroups assigned for PneumoKITy CTVdb

Ctv.db genogroup / folder name	Predicted phenotypes determinable in genogroup	Comment
6A_6B_6C_6D	6A/6B, 6C/6D, serogroup _6(6E)	Genogroup less specific than suggested by distance evaluations to capture large amount of variation observed in serogroup 6 CPS operon and presence of 6E variant.
12F_12A_12B_44_46	12F, 12A/12B/44/46	Serotype 12F can be separated from the others
15F_15A	15F, 15A	
19A_19AF	19A, 19F	Include second stage detection of 19A-like 19F variants (variant wzy*) and separate from 19A phenotype
25F_25A_38	25F/25A, 38	Serotype 38 can be separated from the others

∗*wzy*, gene coding for polysaccharide polymerase.

### Updating the CTVdb with new references and reconfiguring stage 2 determinations

To increase sensitivity to some of the serotypes, DNA sequences spanning the capsular operons of the serotype 18F, 24F and 33F isolates were extracted from the GenBank files created by Prokka annotation of the DNA assemblies from the two representative isolates.

For serotype 24F, the DNA sequence from the first nucleotide of the first codon of the regulatory gene *wzg* to the last nucleotide of the last codon of *rmlD* (which codes for a dTDP-4-dehydrorhamnosereductase, a component of the TDP-Rha synthesis pathway) produced a DNA sequence of length 20 793 bp. When aligned to the original PneumoCaT reference for 24F, the new sequence was nine nucleotides longer, representing three extra codons (coding for V, L, I) at the beginning of *wzg*, but otherwise was similar to the original reference strain with only 234/20793 (1.1 %) variable sites.

For serotype 33F the DNA sequence from the first nucleotide of the first codon of *wzg* to the last nucleotide of the last codon of *wcyO* (which encodes an acetyltransferase) produced a DNA sequence of length 15 931 bp. No *wcjE* gene (which also encodes an acetyltransferase) was found in the annotation, which is present in the reference sequence, instead *wcyO* appears the end of the CPS operon, the *wcyO* ORF appears to be intact and was annotated as a putative acetyltransferase. When aligned to the original PneumoCaT reference for 33F, the new sequence had 251 additional nucleotides at the beginning of the sequence and 931 additional nucleotides at the end. Within the aligned sequence there were 351 variable sites. Due to existing descriptions of a 33F1 variant possessing an inactive *wcyO* we designated this sequence as 33F2 in line with this work [12].

For serotype 18F the DNA sequence from the first nucleotide of the first codon of *wzg* to the last nucleotide of the last codon of *rmlD* produced a DNA sequence of length 20 077 bp. When aligned to the original PneumoCaT reference for 18F, the new sequence had 269 additional nucleotides at the beginning of the sequence and was 932 nucleotides shorter at the end due to non-inclusion of a short hypothetical sequence at the end of the operon. Within the aligned sequence there were 795 variable sites.

The CTVdb was updated to add the new 24F2 and 33F2 sequences to be interpreted correctly in the database. As the 18F reference was so different to the existing reference and was created from an isolate of the SSI Diagnostica type strain, the new 18F sequence directly replaced the original 18F sequence in the reference.fasta.

After the CTVdb update PneumoKITy was then potentially able to determine 54 (58 %) of the 93 serotypes currently distinguishable by the commercially available SSI Diagnostica serotyping sera.

### Results from SSI reference panel FastQ and assemblies

PneumoKITy V1.0 was used to test the 91 reference FastQ pairs obtained from the reference isolates (see Table S4, in pure run mode.

Compared to the expected serotype or genogroup for the 91 reference strains, there was only one unexpected result, a serotype 7C strain reported as mixed with 7B/40. There is a possibility this was contaminated, as the 7C and 7B strains were next to each other in the original sequencing plate. The mixture was estimated at 38.1 % 7B_40 and 61.9 % 7C by use of the median-multiplicity metric in PneumoKITy, a result which was scored as ‘incorrect’ for the purposes of this analysis. Four further samples resulted with the correct serotype or genogroup hit but were flagged AMBER by PneumoKITy, two of these represented low hits at stage 1 screen, these were a serotype 19A and 6C which hit at 88.3% and 87.2% but were otherwise correctly typed as 19A and 6C/6D respectively. The next two samples were 25A and 25F and the AMBER flag was due to a low median multiplicity for the isolates meaning that the multiplicity for the k-mers in the *wcyV* in stage 2 gene presence or absence analysis was below the cut-off of 10, which meant that gene-absence was not scored and the group 25A_25F_38 was returned instead of separating 25F/25A from serotype 38. The overall hit accuracy was 88/91 (97.8 %) to correct result or 90/91 (98.9 %) to genogroup level using the FastQ input.

Compared with FastQ input, the results from running PneumoKITy in pure run mode when the assembly files were used as the input gave only one unexpected result, the 25A isolate resulted as ‘Poor sequence quality’, with a RED RAG score. The top hits were 25A at only 20.6 % and 25F at only 21 %. In addition, three other samples gave AMBER RAG status, to warn of potential problems with the data due to lower than 90 % initial hits. These, however, resulted in the correct call to serotype or genogroup. The assembly statistics for these samples compared to a sample giving a GREEN RAG status are shown in Table 4.

**Table 4.** PneumoKITy metrics and assembly statistics for reference isolate assemblies exhibiting RED or AMBER PneumoKITy result flags, plus an example of a GREEN flagged result. Pure culture run mode

Sample	Expected serotype	Top hit %	RAG status	No. of contigs	L50	N50
**ERR1437928**	10F	100	GREEN	57	9	78 340
**ERR1437950**	19A	88.3	AMBER	68	12	59 981
**ERR1438004**	6C	87	AMBER	71	12	65 406
**ERR1438011**	9L	72.3	AMBER	119	22	24 978
**ERR1437965**	25A	21	RED	79	13	53 365

In general, the PneumoKITy maximum top hit percentage decreased with increasing number of contigs in the assembly (indicating decreasing assembly quality). The RED score for the 25A assembly appeared to be due to the capsular operon genes being broken up between different contigs in the assembly.

The 7C isolate assembly resulted without error as 7C, not mixed as when the FastQ were run. This was due to the hit to the 7B and 7C references being below the 90 % hit cut-off for the assembly input. For the assembly files the accuracy was also 90/91 (98.9 %).

No samples caused errors in the PneumoKITy software run. Data and results from this analysis are available in Table S4.

### Results from final validation with PneumoCaT1 validation set

To validate the PneumoKITy V1.0, the tool was run in pure culture mode on FastQ genome pairs and assemblies created from the original PneumoCaT validation set data. In total 2053 FastQ pairs and assemblies of these FastQ pairs were tested, the data are shown in Table S2. A summary of these results is seen in Table 5.

**Table 5.** Summary results from testing of PneumoCaT1 validation panel samples using PneumoKITy with a) FastQ input and b) assembly input, compared to original results. Pure culture run mode

a) FastQ input			
**RAG status**	**Mismatch**	**Match**	**Grand total**
**AMBER**	6	36	42
**GREEN**	2	1976	1978
**RED**	9	24	33
**Grand total**	**17**	**2036**	**2053**
**b) Assembly input**			
**RAG status**	**Mismatch**	**Match**	**Total**
**AMBER**	4	40	44
**GREEN**	3	1966	1969
**RED**	16	24	40
**Grand total**	**23**	**2030**	**2053**

Several discrepancies between the original published PneumoCaT serotype designation of 12B and PneumoKITy designation of 12F were seen, however this was due to inaccurate original serotyping leading to mis-development of the original PneumoCaT CTVdb and erroneous calling of 12F as 12B, this was fixed in a later release of PneumoCaT (see https://github.com/phe-bioinformatics/PneumoCaT/releases/tag/v1.1) due to this the PneumoKITy calls of 12F for these samples were scored as correct.

For FastQ and assembly input data respectively, 2036 (99.0 %) and 2032 (98.98 %) of the samples gave the expected results compared to the original PneumoCaT 1.0 calls (serotype, genogroup or acapsular call) with either a ‘GREEN’ or ‘AMBER’ RAG status flag or a ‘RED’ status flag where the sample was originally designated as acapsular or if the original PneumoCaT result was ‘Failed’.

Mismatched data are summarised in Table 6.

**Table 6.** Mismatched results from testing of PneumoCaT1 validation panel samples using PneumoKITy compared to original results. NTR=Non-typeable rough

Sample ID	Lab serotype	PneumoCaT serotype	PneumoKITy FastQ result	FastQ RAG status	PneumoKITy assembly result	Assembly RAG status
**ERR1440723**	37	37	Below 70 % hit – Poor Sequence quality, variant or non-typeable organism.	RED	Below 70 % hit – Poor Sequence quality, variant or non-typeable organism.	RED
**ERR1440855**	37	37	Below 70 % hit – Poor Sequence quality, variant or non-typeable organism.	RED	Below 70 % hit – Poor Sequence quality, variant or non-typeable organism.	RED
**ERR1440332**	06A	06A	Serotype within 6A_6B_6C_6D unexpected variant pattern	RED	Serotype within 6A_6B_6C_6D unexpected variant pattern	RED
**ERR1440637**	06A	06A	Serotype within 6A_6B_6C_6D unexpected variant pattern	RED	Serotype within 6A_6B_6C_6D unexpected variant pattern	RED
**ERR1440020**	06C	06C	Serotype within 6A_6B_6C_6D unexpected variant pattern	RED	Serotype within 6A_6B_6C_6D unexpected variant pattern	RED
**ERR1438860**	06D	06D	Serotype within 6A_6B_6C_6D unexpected variant pattern	RED	Serotype within 6A_6B_6C_6D unexpected variant pattern	RED
**ERR1439179**	18C	18A	Mixed serotypes – {'18A', '18C/18B'}	AMBER	Below 70 % hit – Poor Sequence quality, variant or non-typeable organism.	RED
**ERR1439638**	33B	33B	33D	GREEN	33D	GREEN
**ERR1440439**	35F	35F	34	GREEN	34	GREEN
**ERR1438800**	NTR	Failed	25F_25A_38	RED	25F_25A_38	RED
**ERR1438809**	38	38	25F_25A_38	RED	25F_25A_38	RED
**ERR1440458**	38	38	25F_25A_38	RED	25F_25A_38	RED
**ERR1440125**	23F	Failed	23B	AMBER	23B	AMBER
**ERR1440391**	23F	Failed	23B	AMBER	23B	AMBER
**ERR1440598**	NTR	Failed	14	AMBER	14	AMBER
**ERR1439282**	8	8			Below 20 % hit – possible acapsular organism, check species identity and sequence quality.	RED
**ERR1440445**	31	31			Below 70 % hit – Poor Sequence quality, variant or non-typeable organism.	RED
**ERR1439965**	06C	06C			Below 70 % hit – Poor Sequence quality, variant or non-typeable organism.	RED
**ERR1439040**	11A	11A			Below 70 % hit – Poor Sequence quality, variant or non-typeable organism.	RED
**ERR1438836**	12F	12F			12F_12A_12B_44_46: wciI not found in isolate, possible variant	AMBER
**ERR1439960**	15A	15A			Below 70 % hit – Poor Sequence quality, variant or non-typeable organism.	RED
**ERR1440661**	15B	15B			Below 70 % hit – Poor Sequence quality, variant or non-typeable organism.	RED
**ERR1440611**	15C	Mixed: ['15A', '15B/C']			15B/15C	GREEN
**ERR1440740**	10F	Failed	25F_25A_38	AMBER		
**ERR1439227**	15B/C	15B	Mixed serotypes– {'15B/15C', '15F_15A'}	AMBER		

Seven isolates (0.34 %) gave the correct results to genogroup level in the FastQ analysis, with problems detected at stage 2 analysis, *wciN* allele detection at stage 2 for four serogroup 6 isolates failed due to hit percentages just below the 90 % cut-off. In addition, low hit percentage for *wcyD* for distinction of serotype 38 from 25F/25A. Similar problems with stage 2 analysis were also seen in the assembly data with four serogroup 6 isolates also giving similar hits just under the 90 % cut-off for the *wciN* analysis at stage 2 and three serotype 38 isolates also giving a low percentage hit to *wcyD*. Two serotype 37 isolates gave a low hit to the initial *tts* reference below the 70 % cut-off in both the FastQ and assembly data.

Six further isolates gave discrepant results (in both FastQ and assembly results) with either differing serotypes compared to PneumoCaT or where PneumoCaT had failed, a differing serotype compared to the original laboratory type. These were a serotype 33D when PneumoCaT resulted as 33B, the 33D hit was 90 % and 33B was 89 % and as no genogroup was assigned to this type, the top hit result was returned as the second hit was just below the 90 % threshold, which would have resulted in a mixed serotype call. This could be due to a variant serotype as in the reference dataset, 33D was very much lower at 10 % hit when compared to the 33B sample. The second serotype discrepancy was a serotype 34 when PneumoCaT originally resulted as 35F. In the FastQ data serotype 34 hit at 93 % and 35F at only 28.89 % and in the assembly data 34 was 92.7 % and the 35F serotype did not register in the top five hits (lowest hit in top five was 27.7 %). This may have been due to a sample/data mix up, repeating the file in PneumoCaT 1.2 resulted as a failure but with a serotype 34 top hit at 84.6 %, with 35F only giving 30.85 % coverage. A further two isolates resulted as 23B when PneumoCaT failed and original serotyping was 23F, both isolates had top hits below the initial 90 % in both sets of data indicating either poor sequence quality or variant sequences. In the FastQ data, the 23B hit was 82.7% and 84.5 % for both samples with the 23F hit at 65.8% and 64.0% respectively. In the assembly data the 23B hit was 84.5% and 79.9 % with 23F at 63.6% and 62% respectively. This could indicate mixed reads between a 23F and 23B but due to both being within the same genogroup PneumoKITy cannot distinguish them as a mixture. The final two serotype discrepancies were two isolates that were serotype 14 and group 25F/25A/38 respectively when PneumoCaT failed and original phenotypic serotyping was non-typeable.

One serotype 8 isolate failed with a <20 % hit in the assembly data (top hit was to serotype 36 with only 14.9 %). However, this isolate resulted correctly as serotype 8 with 98 % hit in the FastQ data. The N50 and L50 statistics for the assembly were poor at 3227 and 192 respectively.

For the discrepancies where the FastQ data gave a match, but the assemblies had results falling below the 70 % hit threshold, the assembly statistics were as shown in Table 7. Full PneumoKITy hit and stage 2 statistics are contained in Table S2.

**Table 7.** Hit and assembly statistics for isolates which gave a match result in PneumoKITy using FastQ input but failed with <70 % hit using assembly input

Sample id	Lab serotype	PneumoCaT	PneumoKITy assembly result	Top hit %	L50	N50
**ERR1440445**	31	31	Below 70 % hit – Poor Sequence quality, variant or non-typeable organism.	31 44.4 %	14	47 781
**ERR1439965**	06C	06C	Below 70 % hit – Poor Sequence quality, variant or non-typeable organism.	6D 56.9%	15	40 725
**ERR1439040**	11A	11A	Below 70 % hit – Poor Sequence quality, variant or non-typeable organism.	11F 65.3%	25	22 892
**ERR1439960**	15A	15A	Below 70 % hit – Poor Sequence quality, variant or non-typeable organism.	15A 45.5%	16	44 976
**ERR1440661**	15B	15B	Below 70 % hit – Poor Sequence quality, variant or non-typeable organism.	15B 59.7%	16	48 254

One sample resulted as ‘Mixed serotypes- {'18A', '18C/18B'}” in PneumoKITy FastQ data and as a below 70 % hit in the assembly data. This isolate gave an 18A result in PneumoCaT, but the laboratory serotype result was 18C. Potentially this isolate could be either mixed or a serotype variant with sequence characteristics of both serotypes.

One isolate was scored as mismatch in the assembly data due to the original sample being mixed (15A and 15B/C) in PneumoCaT. However, it was resulted as 15B/C which correctly matched with the original laboratory serotype 15C.

Sample ERR1440740 gave completely discrepant results with differing top hits between the FastQ and assembly data. The original PneumoCaT result was ‘Failed’ for this sample and the lab serotype was reported as 10F. The assembly data reported a <20 % hit result with a top hit of 10C at only 11.1 %, while the FastQ data returned a 99.3 % hit with serotype 38. However, the maximum median-multiplicity metric was only 15, which indicated poor depth of read data in this sample resulting in poor multiplicity of k-mers in the analysis. For comparison, the average maximum median-multiplicity metric for the top hits in the full set of FastQ data from the validation panel was 114.45.

The final discrepancy was mixed serotypes detected with PneumoKITy for a sample which had original PneumoCaT result 15B/C. PneumoKITy reported the estimated mixture of the isolates to be 15B/15C 53.47 % and 15F_15A 46.53 %, the top hits were 15C 95.7 %, 15B 95.6 %, 15A 91.4 %, and 15F 69.5 %,

### Results from final validation with nasopharyngeal culture genome set

For final validation of PneumoKITy V1.0, the tool was run in pure culture mode on FastQ genome pairs and assemblies created from isolates obtained from a study of pneumococcal nasopharyngeal carriage [23]. In total 876 FastQ pairs and assemblies of these FastQ pairs were tested (Table S3) In this carriage study these samples were isolated as single colony picks and therefore considered as ‘pure culture’ isolates rather than potentially mixed (e.g., plate sweep samples). The summary results are shown in Table 8.

**Table 8.** Summary results from testing of nasopharyngeal carriage panel isolates using PneumoKITy with a) FastQ input and b) assembly input, compared to original PneumoCaT results

a) FastQ input			
**RAG status**	**Mismatch**	**Match**	**Grand total**
**AMBER**	3	3	6
**GREEN**	1	824	825
**RED**	7	38	45
**Grand total**	**11**	**865**	**876**
**b) Assembly input**			
**RAG status**	**Mismatch**	**Match**	**Total**
**AMBER**	1	11	12
**GREEN**	0	816	816
**RED**	10	38	48
**Grand total**	**11**	**865**	**876**

For FastQ and assembly input data respectively, 865 (98.7 %) of the samples gave the expected results compared to the original PneumoCaT 1.0 calls (serotype, genogroup or acapsular call) with either a ‘GREEN’ or ‘AMBER’ RAG status flag or a ‘RED’ status flag where the PneumoCaT result was ‘Failed’ which had been originally designated as NT (non-typable).

Mismatched data are summarised in Table 9. One sample that had given a non-typeable result by PneumoCaT, gave a serotype 23B result and had an MLST sequence type and GPSC (439 and 7 respectively), indicative of serogroup 23 in the original study.

**Table 9.** Mismatched result data for the nasopharyngeal carriage isolate set

Sample ID	PneumoCaT serotype	PneumoKITy FastQ result	FastQ RAG status	PneumoKITy assembly result	Assembly RAG status
ERR3530897	nt	23B	GREEN	23B	AMBER
ERR3530844	6A	6A_6B_6C_6D	AMBER	6A_6B_6C_6D	RED
ERR3530272	6C	6A_6B_6C_6D	RED	6A_6B_6C_6D	RED
ERR3530449	6A	6A_6B_6C_6D	RED	6A_6B_6C_6D	RED
ERR3530750	6A	6A_6B_6C_6D	RED	6A_6B_6C_6D	RED
ERR3530848	6A	6A_6B_6C_6D	RED	6A_6B_6C_6D	RED
ERR3530980	6A	6A_6B_6C_6D	RED	6A_6B_6C_6D	RED
ERR3531026	6A	6A_6B_6C_6D	RED	6A_6B_6C_6D	RED
ERR3531108	6A	6A_6B_6C_6D	RED	6A_6B_6C_6D	RED
ERR3530382	15A	Mixed serotypes- {'23B', '15F_15A'}	AMBER		
ERR3530324	6B	6A_6B_6C_6D	AMBER		
ERR3530801	6C			6A_6B_6C_6D	RED
ERR3530903	23A			Below 70 % hit – Poor Sequence quality, variant or non-typeable organism.	RED

Most discrepancies in this set were serogroup 6 isolates which resulted as the serogroup rather than a more specific 6 A/C or 6C/D result due to failure of the second stage *wciN* determination to hit at >90 % or in one case due to the median multiplicity of the k-mers screening against *wciN* being below the cut-off of 10.

For one sample PneumoKITy detected a serotype mixture of 15A/15F with 23B which had not been detected in the original PneumoCaT run, the original PneumoCaT result was 15A. The final discrepancy was the failure of a 23A serotype to reach the initial 70 % screen lower cut-off in the assembly data, though the sample resulted correctly in the FastQ data.

### Detection of multiple serotypes in mixed samples

To evaluate the capacity of PneumoKITy to detect multiple serotypes in mixed samples, the tool was run on 52 publicly available mixed serotype sequence samples prepared by Knight *et al*, [11] consisting of different combinations of serotypes 1, 3, 4, 6B, 7F, 9V, 14, 18C, 19F and 23F (Table S5) and compared with SeroCall. One sample (SRR9998155) had no serotypes detected by either method, but the FastQ files only had 3250 reads, suggesting there was not sufficient sequence coverage to detect the serotypes in the sample. This sample was excluded from further analysis.

A comparison of multiple serotype detection of PneumoKITy and SeroCall on these samples is summarized in Table 10. PneumoKITy correctly detected all expected serotypes in the sample in 47/51 (92.2 %) to either type (1, 3, 4, 14, 19F, 23F) or group (6A/6B/6C/6D, 7F/7A, 9A/9V, 18B/18C) level compared with 42/51 (82.4 %) by SeroCall.

**Table 10.** Summary results of multiple serotype detection of mixed cultures of PneumoKITy compared with SeroCall. A match was defined as all expected serotypes and no unexpected serotypes were detected in the sample

Method	Mismatch	Match	Grand total
PneumoKITy	4	47	51
SeroCall	9	42	51

Table 11 lists the samples where the serotypes determined by either PneumoKITy or SeroCall did not match what was expected. SRR9998215 was a mixture of serotypes 18C and 19F, however both PneumoKITy and SeroCall detected a third serotype (23F). Given the relative abundance of 23F was ~9 %, this result likely represents contamination of the culture with a 23F strain.

**Table 11.** Discrepant serotype results by PneumoKITy and/or SeroCall of mixed culture samples. Relative abundance is noted in parentheses where relevant. Note: as PneumoKITy will type 18B or 18C and 9V or 9A to group level, calls of 18B and 9A made by SeroCall were considered ‘correct’ for fair comparison to PneumoKITy

Sample ID	Expected mix	SeroCall result	PneumoKITy result	RAG status	Mismatch description
SRR9998215	18C, 19F	18C, 19F, 23F (8.96 %)	18C/18B, 19F, 23F (10.2 %)	GREEN	23F detected by both methods
SRR9998218	1, 3, 4, 6B, 7F, 9V, 14, 18C, 19F, 23F	1, 3 (2.7 %), 4, 7F, 9V, 14, 18B, 19F, 23F	1, 4, 6A/6B (19.69 %), 7F/7A, 9A/9V, 14, 18C/18B, 19F, 23F	GREEN	Serotype 3 not detected by PneumoKITy 6B not detected by SeroCall
SRR9998177	3, 6B, 7F, 9V, 14	3 (1.5 %), 6B, 7F, 9V, 14	6A/6B, 7F/7A, 9A/9V, 14	GREEN	Serotype 3 not detected by PneumoKITy
SRR9998166	1, 3, 4, 6B, 7F, 9V, 14, 18C, 19F, 23F	1, 3 (0.5 %), 4, 6B, 7F, 9V, 14, 18B, 19F, 23F	1, 4, 6A_6B_6C_6D, 7F/7A, 9A/9V, 14, 18C/18B, 19F, 23F	GREEN	Serotype 3 not detected by PneumoKITy
SRR9998157	3, 6B, 7F, 9V, 14	3, 4 (1 %), 6B, 7F, 9A, 14	3, 6A_6B_6C_6D, 7F/7A, 9A/9V, 14	GREEN	Serotype 4 detected by SeroCall
SRR9998185	1, 19F	1, 4 (0.4 %), 19F	1, 19F	GREEN	Serotype 4 detected by SeroCall
SRR9998188	3, 6B, 7F, 9V, 14	3, 4 (0.6 %), 6B, 7F, 9V, 14	3, 6A/6B, 7F/7A, 9A/9V, 14	GREEN	Serotype 4 detected by SeroCall
SRR9998190	3, 6B, 7F, 9V, 14	3, 4 (0.7 %), 6B, 7F, 9V, 14	3, 6A/6B, 7F/7A, 9A/9V, 14	GREEN	Serotype 4 detected by SeroCall
SRR9998199	19F, 23F	1 (0.3 %), 4 (0.3 %), 19F, 23F (10.2 %)	19F, 23F (8.96 %)	GREEN	Serotypes 1 and 4 detected by SeroCall
SRR9998219	3, 6B, 7F, 9V, 14	3, 4 (0.7 %), 6B, 7F, 9V, 14	3, 6A/6B, 7F/7A, 9A/9V, 14,	GREEN	Serotype 4 detected by SeroCall
SRR9998222	18C, 19F	4 (0.4 %), 18B, 18C, 19F, 23F (0.3 %)	18C/18B, 19F	GREEN	Serotypes 4, and 23F detected by SeroCall
SRR9998155 - EXCLUDED	3, 6B, 7F, 9V, 14	No result	No result	RED	Low sequence coverage

Compared with SeroCall, PneumoKITy failed to detect serotype 3 in three samples. For these samples the serotype 3 hit percentage was below 90 % threshold (82.6%, 73.2% and 69.7 %) and the relative abundance of serotype 3 in these samples as determined by SeroCall were less than 3 %.

For the remaining seven discrepant samples, SeroCall detected a serotype that was not expected to be present in the sample. All these serotype calls had a relative abundance of less than 1%, suggesting these may represent contamination of these samples during the DNA extraction procedure.

To assess the ability of PneumoKITy to determine the relative abundance of serotypes in a sample, samples mixed in known proportions prepared by Knight *et al*. [11] were compared with the relative abundance calculated by PneumoKITy and SeroCall (Table 12). Both methods performed well in estimating the relative abundance of each of the serotypes within each sample. PneumoKITy was more accurate at estimating the relative abundance with an average estimate of 1.5 % deviation from the expected abundance compared with 2.2 % for SeroCall.

**Table 12.** Comparison of relative abundance of each serotype detected by PneumoKITy or SeroCall in samples mixed in known ratios

		Relative abundance of serotype (%)				
**Sample ID**		**1**	**4**	**18C**	**19F**	**23F**
SRR9998169	Known mixture	–	–	–	50.00	50.00
	PneumoKITy	–	–	–	49.81	50.19
	SeroCall	–	–	–	49.00	51.00
SRR9998160	Known mixture	–	–	–	33.00	67.00
	PneumoKITy	–	–	–	32.82	67.18
	SeroCall	–	–	–	31.10	68.90
SRR9998161	Known mixture	–	–	–	20.00	80.00
	PneumoKITy	–	–	–	20.90	79.10
	SeroCall	–	–	–	17.40	82.60
SRR9998159	Known mixture	–	–	–	11.00	89.00
	PneumoKITy	–	–	–	11.60	88.40
	SeroCall	–	–	–	10.30	89.70
SRR9998156	Known mixture	–	–	–	9.00	91.00
	PneumoKITy	–	–	–	9.57	90.43
	SeroCall	–	–	–	8.70	91.30
SRR9998197	Known mixture	–	–	–	1.00	99.00
	PneumoKITy	–	–	–	1.30	98.70
	SeroCall	–	–	–	0.90	99.10
SRR9998191	Known mixture	17.00	–	–	17.00	66.00
	PneumoKITy	10.53	–	–	18.42	71.05
	SeroCall	9.00	–	–	16.50	74.60
SRR9998154	Known mixture	40.00	20.00	10.00	10.00	20.00
	PneumoKITy	45.28	19.44	4.44	10.83	20.00
	SeroCall	44.80	19.90	3.70	9.20	22.40

### Benchmarking

PneumoKITy V1.0, PneumoCaT V1.2.1 and SeroCall were run on serotype 1 (ERR1437924) and serotype 15A (ERR1437939) SSI reference FastQ files in pure culture mode. The full data on mean run time and memory requirement from three runs are recorded in Table S6. PneumoKITy was 18× faster and used over 10× less memory than PneumoCaT and 25× faster and used 1.9× less memory than SeroCall on a serotype 1 isolate (stage 1 only) using a single thread. On a 15A genome using a single thread, PneumoKITy was 4.4× faster and used 9× less memory than PneumoCaT and 5.9× faster and used 1.9 × less memory than SeroCall. The memory requirement for PneumoKITy was consistent between the two serotype runs remaining at around 88 Mb. Increasing the number of threads to four reduced the run time for PneumoKITy from 7.96 s to 5.89 s for the serotype 1 compared to PneumoCaT and SeroCall both resulted in just under 1 min when four threads were used for the analysis compared to 2.28 min and 3.25 min respectively for a single thread.

PneumoKITy was then run with the genome assembly files for serotype 1 and 15A in pure culture mode. SeroCall and PneumoCaT 1.2.1 cannot run from assemblies so were not compared. Determinations from assemblies in pure culture mode for both serotype 1 (stage 1 only) and serotype 15A (stage 2) were completed in less than 1 smeg and using fewer than 90 megabytes of memory using PneumoKITy.

## Discussion

Sensitive detection of multiple pneumococcal serotypes in nasopharyngeal carriage samples would give valuable additional information to inform vaccine implementation and surveillance of *S. pneumoniae* disease. We have developed PneumoKITy a lightweight, fast and accurate option for determining multiple serotypes present in genomic read data (FastQ).

Mash Screen, as used in PneumoKITy, was found to be a highly effective method of determination of serotype from WGS data. The method was specific enough to determine genogroups that were similar to those defined for the Bowtie two mapping approach used for PneumoCaT [5]. In some cases, Mash Screen [13] was found to be more specific than the original mapping method, meaning that serotypes that fall in a genogroup in PneumoCaT can be determined fully with a stage 1 screen alone (e.g. serotypes in serogroup 10), in total PneumoKITy can determine 54 (58.1 %) of the 93 serotypes in the SSI Diagnostica phenotypical serotyping scheme to type level with the remainder called to serogroup or sub-serogroup level (e.g. 11A/D)

Mash Screen was successfully implemented as stage 2 analysis method for genogroups where the difference can be determined using gene presence/absence or alleles of genes. Serotypes 19A, 19AF (19A-like CPS operon background with variant *wzy* and phenotype 19F [24]), 15A and 15F can be fully determined in this way and some genogroups can be sub-divided into serotypes and sub-serogroups, e.g. serotype 12F can be subdivided from sub-serogroup 12A/12B/44/46; serogroup 6 can be sub-divided into 6A/6B and 6C/6D with subtype 6E also being detected and reported as 6E_serogroup6 to reflect that the phenotype of the genetic type 6E isolate within serogroup 6 is not possible to derive using PneumoKITy.

PneumoKITy reports the expected phenotype of the organism as if it had been determined using the commercially available typing sera (SSI Diagnostica, Copenhagen). However, by examination of the top-hits and other information included in the PneumoKITy output – the genetic type of the organism can be suggested, for example genetic subtype 23B1 discovered in the initial development of PneumoCaT [12] will result as expected phenotype 23B. However, the top hit in the stage 1 analysis will be to the 23B1 reference rather than the 23B reference, in this way the similarity of the isolate CPS operon to previously published subtypes of serogroup 19 and 6 [16] can be determined by examination of the top hits.

PneumoKITy incorporates a newly designed SQL CTVdb which is central to the software and used for all serotype result interpretations. PneumoKITy provides useful outputs that are more comprehensive and user friendly than the outputs provided by PneumoCaT and employs a helpful RAG status flag to alert the user to the quality of the results. The software outputs a human-readable text report containing the results, RAG status and quality system data (file locations, software versions etc.), csv files suitable for computational manipulation, if necessary, plus full analysed Mash Screen result output csv for the capsular operon screen (and any subsequent gene screens). Outputs are also tailored depending on whether pure run mode is selected, or mix run mode is selected. If pure mode is selected, mixed serotypes can still be detected with lower sensitivity, but any serotypes in the mixture that require subtyping at stage 2 are not subtyped, though an estimation of the abundance of the genogroups determined is still given. In mix mode subtyping of all the serotypes detected in the mix is attempted if applicable.

Analysis of the reference isolates and the carriage study data showed PneumoKITy to be highly accurate. Its sensitivity to mixtures when used in mix mode is useful for detection of multiple serotypes where mixtures might be expected, for example if testing sweep cultures from carriage samples or as a means of quality control of DNA extraction procedures.

Some areas for serotype specificity improvement were noted in the data, for example in the pure culture nasopharyngeal carriage set, several serogroup 6 samples resulted as 6A/6B/6C/6D rather than the expected sub-groups 6A/6B or 6C/6D. This was due to the second stage detection of *wciN* giving hits just below the hit cut-off of 90 %. Although in this set 111 samples did correctly type as the more specific 6A/6B or 6C/6D sub-serogroup, indicating the problem was not widespread, this did suggest that the stage 2 gene-based analysis was either set with too-strict a cut-off or would be better served by inclusion of more than one gene version for each detected gene in the analysis, the latter being the preferable solution.

The reference sequences used in the original PneumoCaT CTVdb are from very historical strains and therefore may not reflect the sequences of the currently circulating strains. Due to the extra specificity of the Mash Screen k-mer-based method, this results in lower hit percentage due to removal of non-exact match k-mers than would happen in the mapping-based method used by PneumoCaT, and this is particularly emphasised in the gene-based analysis where the percentage hit is much more affected by loss of individual k-mers, due to one nucleotide difference, than in longer sequences. Additional references were added to the PneumoKITy CTVdb stage 1 capsular operon reference to reflect expected diversity of sequences, with inclusion of capsular operon references for defined subtypes of serogroups 6 and 19 from Elberse *et al.* [16] and new capsular operon references were created and added to the stage 1 references during the development of PneumoKITy for serotypes 18F, 33F and 24F.

For 33F it was found that the isolates that gave an issue and caused an additional reference sequence to be necessary, had a missing gene *wcjE*, which had been replaced with *wcyO* in the capsular operon sequence. Variants similar to these had been noted in previous work [25]. It is highly likely that additional variants and representative capsular operon references will need to be added to the CTVdb, for other serogroups in stage 1 which demonstrated higher percentage of samples hitting with AMBER RAG status due to the initial hit being below 90 %.

No additional references were added to the gene sequence references used in stage 2 of the PneumoKITy analysis, and thus AMBER flagged sample results could be upgraded to GREEN by inclusion of additional representatives of the genes or alleles used in stage 2 presence/absence or allele-based analyses.

Analysis of the mixed serotype samples showed that detection of low abundance serotypes in mixtures was possible. PneumoKITy correctly detected 92.2 % of the serotypes in the panel of mixed serotype samples tested and was able to detect a minor serotype with 1 % expected abundance in some samples. The calculated abundance reported by PneumoKITy on samples of known mixtures was highly accurate and estimated the abundance to within 1.5 % of the expected abundance in the mix which was a closer match than found by SeroCall (2.2 %). However, PneumoKITy did present a limitation for detecting serotype 3 in some of the mixtures, with serotype 3 being undetected in three samples. Of all the mixed samples containing serotype 3, these three had the lowest abundance of serotype 3 as determined by SeroCall. The hit percentage for serotype 3 in these samples ranged from 70–83 % (Table S5) suggesting that the abundance of the serotype 3 was too low for the detection using the >90 % hit cut off in the presence of other serotypes with hit percentage above this level. At present PneumoKITy ignores these due to the presence of initial hits above the 90 % default cut-off setting, but if it was the only serotype present the cut-off drop to 70 % could have then detected them. The hit percentage cut off can be manually reduced using the -p flag which would improve the sensitivity for detection of the low abundance samples in this case. Further work could be undertaken to improve the sensitivity for detection in mixtures, perhaps by flagging serotypes that are not in genogroups but that hit with a percentage level between 70 % and the input cut-off or by inclusion of additional references if the current reference is not representative of the serotype so that the initial hit percentage is higher and it is less likely to dip below 90 % when in low abundance.

Some serotypes missed by PneumoKITy in the incubated mixture samples but called by SeroCall were missed due to very low median-multiplicity of the specific k-mers in the mixture in the majority of cases. This could represent very low numbers of original reads in the data for this serotype, which coupled with the sequence specific nature of the k-mer analysis could reduce k-mers included leading to failure to detect. A sensible initial median multiplicity cut-off value of 4 was set for the PneumoKITy mixture analysis which means that each k-mer included in the analysis must be represented four times in the dataset. If this cut-off is reduced further some low abundance serotypes may be called. However, this greatly increases the chances of calling hits with sequence errors or very low-level contaminants. Distinguishing contaminants from genuine serotypes present in the data is difficult, but the information included in the additional files produced by PneumoKITy can be used along with laboratory experience of the extraction and sequencing techniques to judge whether a low-level serotype resulting in the k-mer analysis with below four median-multiplicity could be judged as a true positive. PneumoKITy can run with less stringent median-multiplicity requirement (-n) if it was felt the cut-off was too strict. As PneumoKITy retains all data used for the analysis in additional csv files, results from samples which did not hit the cut-offs can be examined post-run.

An additional feature of PneumoKITy is its ability to run on genome assembly (Fasta) data when run in pure detection mode. This ability was tested by parallel running of the reference and nasopharyngeal carriage data in pure culture detection mode. The use of assembly data gave almost identical results to that of the FastQ input data. However, the quality of the assembly input greatly affected the outcomes; in some cases, the input assembly data was poor with poor L50 and N50 scores and no result was obtained. In other cases, though the L50 and N50 scores of the assemblies were good, no result was obtained. Annotation of these assembly files revealed that the capsular operon in these cases was split over multiple contigs which had affected the assembly input but not the result from the FastQ input.

As with all analytical processes it is best if quality assessment of input data is performed, and results judged accordingly. As PneumoKITy data resulting with the <20 % hit can be assumed to represent an acapsular organism, the quality of the input data must be considered if scoring these results this way. In its current validated form described in this manuscript, PneumoKITy has no ability to determine if a non-capsular pneumococcus might exist in a mixture with a capsular pneumococcus. However, a developmental version of PneumoKITy which has sequences representing non-capsular organisms has been added to a development branch on the PneumoKITy GitHub repository (https://github.com/CarmenSheppard/PneumoKITy/tree/non_cap). This version has not yet been fully validated; users may wish to validate this version for their own purposes. In addition, PneumoKITy does provide an option for users to provide a custom CTVdb database and reference files, so users can run the software against their own reference sequences.

Benchmarking showed PneumoKITy to be extremely fast and with lower memory requirements than PneumoCaT 1.2.1. The determinations were even quicker, resulting in <1 s, when run on assembly files. The software cannot determine final serotype for serotypes that require SNP or functional gene analysis, so the addition of these methods would increase runtime as they would require additional bioinformatic methods for determination rather than k-mer screening. However, PneumoKITy can determine more serotypes in stage 1 than PneumoCaT 1.2.1, and therefore many of the samples that are run in the software will result to a final serotype quickly using the k-mer screen analysis alone. PneumoKITy also demonstrates high accuracy for determination of multiple serotypes in complex samples which should facilitate studies of the serotypes present in nasopharyngeal carriage.

## Supplementary Data

Supplementary material 1

Supplementary material 2

Supplementary material 3

Supplementary material 4

Supplementary material 5

Supplementary material 6

Supplementary material 7
