# Supplementary figures and images for: PneumoKITy: A fast, flexible, specific, and sensitive tool for Streptococcus pneumoniae serotype screening and mixed serotype detection from genome sequence data
Source: Microb Genom. 2022 Dec 14;8(12):mgen000904. doi: 10.1099/mgen.0.000904 (PMC9837567; doi:10.1099/mgen.0.000904)

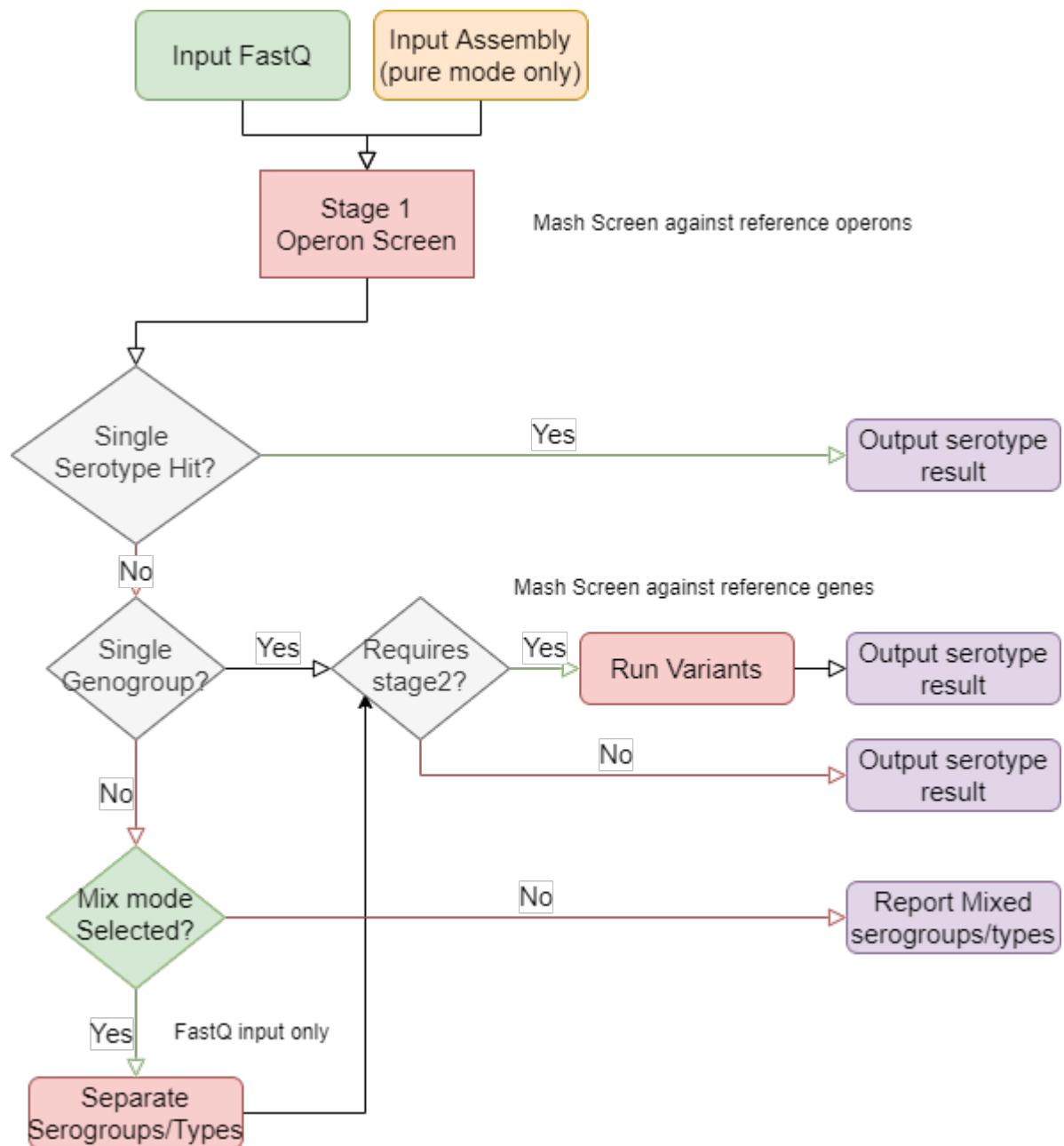

Supplement: Supplementary material 1 [file mgen-8-904-s001.pdf]
