# Supplementary material for: PneumoKITy: A fast, flexible, specific, and sensitive tool for Streptococcus pneumoniae serotype screening and mixed serotype detection from genome sequence data
Source: Microb Genom. 2022 Dec 14;8(12):mgen000904. doi: 10.1099/mgen.0.000904 (PMC9837567; doi:10.1099/mgen.0.000904)
Supplement: Supplementary material 7 [file mgen-8-904-s007.pdf]

Supplementary Table 6 - Comparison of mean run time and memory requirements over 3 runs for PneumoKITy, PneumoCaT 1.2.1 and SeroCall on FastQ files from serotype 1 and serotype 15A isolates (pure culture FastQ analysis mode).

	**Software**	**Wallclock time (min:sec:ms)**	**Memory (megabytes)**
Serotype 1 single thread	PneumoCaT V1.2.1	02:28.87	935.27
	PneumoKITy V1.0	00:07.96	88.43
	SeroCall	03:25.15	169.77
Serotype 1 four threads	PneumoCaT V1.2.1	00:56.48	1052.43
	PneumoKITy V1.0	00:05.89	88.28
	SeroCall	00:58.46	523.39
Serotype 15A single thread	PneumoCaT V1.2.1	01:58.30	797.34
	PneumoKITy V1.0	00:27.21	88.62
	SeroCall	02:39:86	169.82
Serotype 15A four threads	PneumoCaT V1.2.1	00:42.70	798.23
	PneumoKITy V1.0	00:20.66	88.47
	SeroCall	00:47.34	507.77
